# Women’s quality of sleep and in vitro fertilization success

**DOI:** 10.1038/s41598-022-22534-0

**Published:** 2022-10-19

**Authors:** Marco Reschini, Massimiliano Buoli, Federica Facchin, Alessia Limena, Chiara Dallagiovanna, Valentina Bollati, Edgardo Somigliana

**Affiliations:** 1 Infertility Unit, Fondazione IRCCS Ca’ Granda Ospedale Maggiore Policlinico, Via M. Fanti, 6, 20122 Milan, Italy; 2 Department of Neurosciences and Mental Health, Fondazione IRCCS Ca’ Granda Ospedale Maggiore Policlinico, Milan, Italy; 3 Department of Pathophysiology and Transplantation, Università degli Studi di Milano, Milan, Italy; 4 Department of Psychology, Catholic University of the Sacred Heart, Milan, Italy; 5 Department of Clinical Sciences and Community Health, Università degli Studi di Milano, Milan, Italy

**Keywords:** Physiology, Health care

## Abstract

Women undergoing in vitro fertilization (IVF) are emotionally challenged. Anxiety, depression, and sleep disturbances are common complaints. The impact of these symptoms on IVF outcome is however debated. In this study, we aimed at investigating whether sleep quality and psychological health can affect the chances of success of the procedure. Women undergoing IVF were recruited at the time of oocytes retrieval. Women’s sleep quality and psychological health was assessed using the Pittsburgh Sleep Quality Index (PSQI), the Fertility Problem Inventory (FPI), and the Hospital Anxiety and Depression Scale (HADS). Baseline characteristics and results of the three scales were compared between women who did and did not succeed. Overall, 263 women were included, of whom 81 had a clinical pregnancy (31%). As expected, successful women were younger, and their ovarian reserve was more preserved. FPI and HADS scores did not differ. Conversely, a statistically significant difference emerged for the PSQI score, the median [interquartile range] in pregnant and non-pregnant women being 4 [3–5] and 5 [3–7], respectively (*p* = 0.004). The crude and adjusted OR of pregnancy in women with a PSQI > 5 (indicating impaired sleep quality) was 0.46 (95% CI 0.25–0.86, *p* = 0.02) and 0.50 (95% CI: 0.26–0.94, *p* = 0.03), respectively. In conclusion, low sleep quality is common in women scheduled for IVF and could influence the success of the procedure.

## Introduction

Infertility is a prevalent and disabling condition for which couples seek medical care^1^. In vitro fertilization (IVF) is the main type of Assisted Reproductive Treatment (ART) procedure in which oocytes are fertilized outside the female reproductive system^2^. Its use has steadily increased, in part due to its demonstrated safety in terms of perinatal outcome^3,4^.

Infertility is globally recognized as a source of significant psychological distress^5,6^ and the resort to IVF may further deteriorate the mental wellbeing of intended parents^6^. These treatments are emotionally exhausting, and often multiple attempts are needed to achieve a pregnancy^7^. The recent observation that women who conceived with IVF did not show an increased risk of perinatal affective disorders compared to those who conceived naturally is only partly reassuring^8^.

Available data are contrasting with regards to the effect of women’s psychological wellbeing on the outcome of IVF. There is evidence that baseline high levels of stress may be negatively associated with IVF success, as reported by two independent Greek contributions^9,10^. In contrast, studies evaluating the effect of anxiety and depression on IVF outcome are discordant. Three studies (conducted respectively in China, Kazakhstan, Czech Republic) including a total of 601 women showed that severity of depression^11^ and anxiety^11–13^ were associated with lower probability of pregnancy. On the contrary, three studies, conducted respectively in Israel^14^, Greece^15^ and Iran^16^ with a global sample of 397 women, failed to find a significant association between the severity of affective symptoms and the chance of pregnancy. The presence in women of prominent alexithymia, defined as the incapacity to identify and describe emotions^17^, was reported to be associated with higher success rate in a single study^18^. Moreover, preliminary findings suggest the importance of favourable emotional dynamics of couples for a positive IVF outcome^19^.

In recent years, there is a growing interest on sleep quality among infertile women^20–23^. Investigating sleep quality in this setting is particularly attracting because of the significant association with anxiety and stress. In other words, sleep disturbances could be an easily detectable sign of impaired mental health that could be useful in clinical practice. Indeed, identifying couples who are overwhelmed by infertility and its treatments could allow to implement targeted interventions to improve psychological wellbeing^24^. In addition, one may hypothesize that sleep quality could affect IVF success on its own, as it does for natural conceptions, due to the well-established association between sleep and the hormonal system^25^. Evidence on this issue is however scant and inconclusive^20,26^.

In the light of these considerations, the purpose of the present research was to investigate whether the quality of sleep and the psychological health of infertile women at treatment initiation were associated with IVF outcome. Specifically, we hypothesized to find poorer quality of sleep and psychological wellbeing in women who did not become pregnant versus those who were successful.

## Results

**Figure 1:**
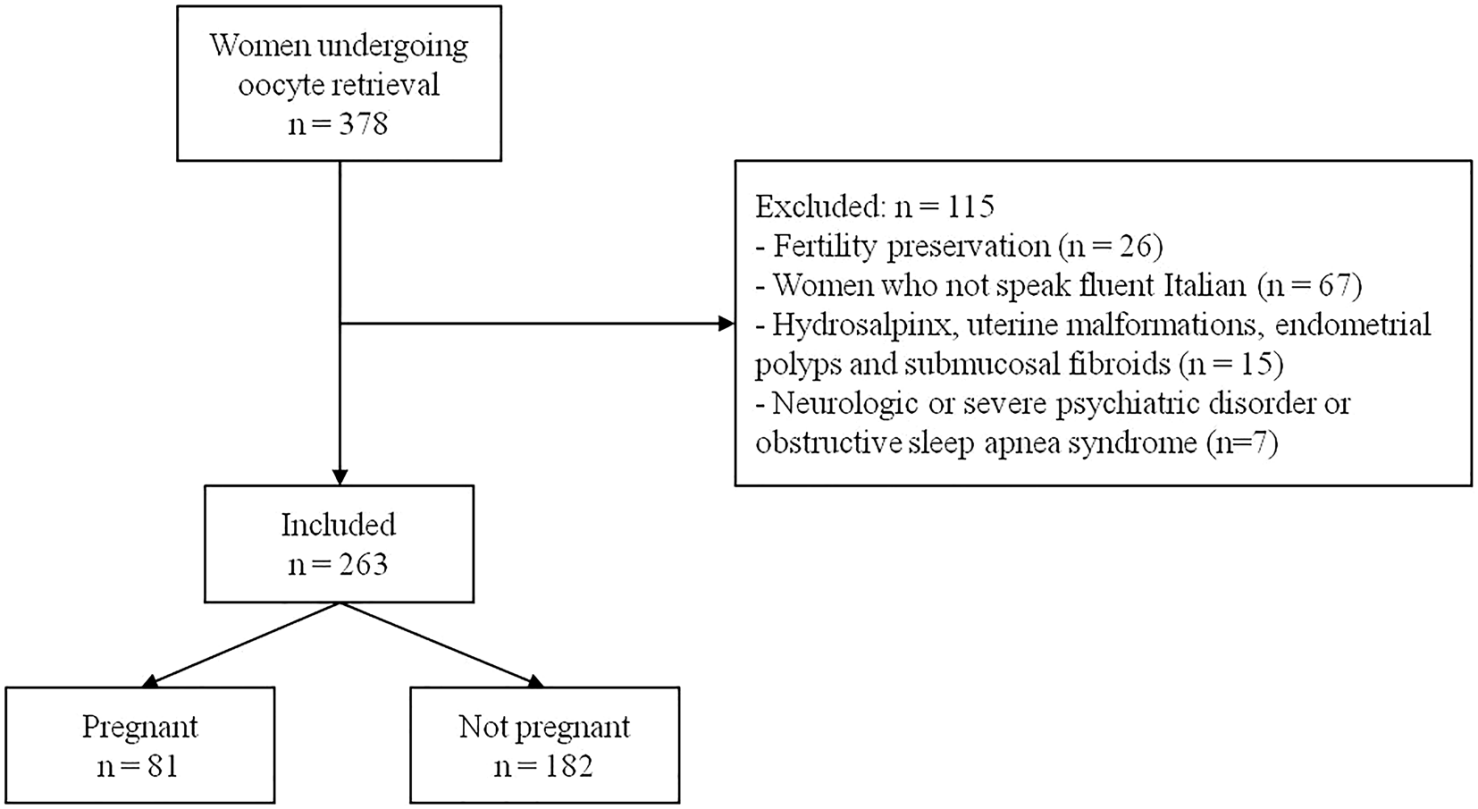
Flow-diagram of the study.

The flow-diagram of the study is shown in Fig. 1. Overall, 263 women were included, of whom 81 had a pregnancy (31%). Baseline characteristics of women who did and did not become pregnant are illustrated in Table 1. As expected, a statistically significant difference emerged for age, showing that women who were successful were younger. Moreover, all biomarkers of ovarian reserve indicated a more preserved condition in women who achieved a pregnancy (lower FSH and higher AMH and AFC). The most significant difference was observed for AFC. No significant differences were detected for education, employment, parity, smoking status and BMI. The causes of infertility did not also differ. The main characteristics of the IVF cycle are shown in Table 2. As expected, all variables significantly differ except for the gonadotropins dose.

**Table 1:** Baseline characteristics of women who did and did not achieve clinical pregnancy.

Characteristics	Successfull conception N = 81	Failed conception N = 182	*p*
Age (years)	35.9 ± 3.7	37.0 ± 4.0	0.05
**Education**			0.64
Primary and middle school	8 (7%)	11 (6%)	
High school	18 (22%)	50 (27%)	
University	57 (71%)	121 (67%)	
**Job status**			0.80
Active	74 (91%)	169 (93%)	
Non active	7 (9%)	13 (7%)	
Smoking	7 (9%)	22 (12%)	0.52
BMI (Kg/m^2^)	21.4 [19.7–24.2]	21.7 [20.0–24.5]	0.48
Duration of infertility (months)	35 [24–48]	30 [24–48]	0.43
Previous deliveries	16 (20%)	36 (20%)	1.00
Serum FSH (IU/mL)	7.3 [5.8–8.7]	7.9 [6.6–9.8]	0.05
Serum AMH (ng/mL)	2.2 [1.2–3.3]	1.8 [0.8–3.0]	0.05
AFC	12 [8–18]	8 [5–13]	0.001
**Indication to IVF**			0.65
Male factor	26 (32%)	58 (32%)	
Endometriosis	9 (11%)	25 (14%)	
Tubal factor (PID)	7 (9%)	11 (6%)	
Anovulation refractory to ovulation induction	3 (4%)	5 (3%)	
Unexplained	26 (32%)	48 (26%)	
Genetic	4 (5%)	8 (4%)	
Mixed	6 (7%)	27 (15%)	

Data is reported as Mean ± SD, Median [IQR] or Number (%) as appropriate.

*BMI* body mass index, *FSH* follicle stimulating hormone, *AMH* anti-mullerian hormone, *IVF* in vitro fertilization, *PID* pelvic inflammatory disease.

**Table 2:** Characteristics of the IVF cycles in women who did and did not achieve clinical pregnancy.

Characteristics	Successfull conception N = 81	Failed conception N = 182	*p*
Total dose of gonadotropins (IU)	1800 [1500–2225]	1800 [1475–2250]	0.67
Duration of stimulation (days)	9 [8–10]	8 [7–10]	0.04
Number of follicles ≥ 15 mm	7 [5–11]	5 [3–7]	< 0.001
Number of oocytes retrieved	7 [4–11]	5 [2–7]	< 0.001
Number of mature oocytes	6 [4–8]	4 [2–6]	< 0.001
Number of embryos	4 [3–6]	2 [1–4]	< 0.001
**Number of embryo transfers**			< 0.001
0	0 (0%)	43 (24%)	
1	55 (68%)	99 (54%)	
2	21 (26%)	31 (17%)	
≥ 3	5 (6%)	9 (5%)	

Data is reported as Median [IQR] or Number (%) as appropriate.

*BMI* body mass index, *FSH* follicle stimulating hormone, *AMH* anti-mullerian hormone, *IVF* in vitro fertilization, *PID* pelvic inflammatory disease.

**Figure 2:**
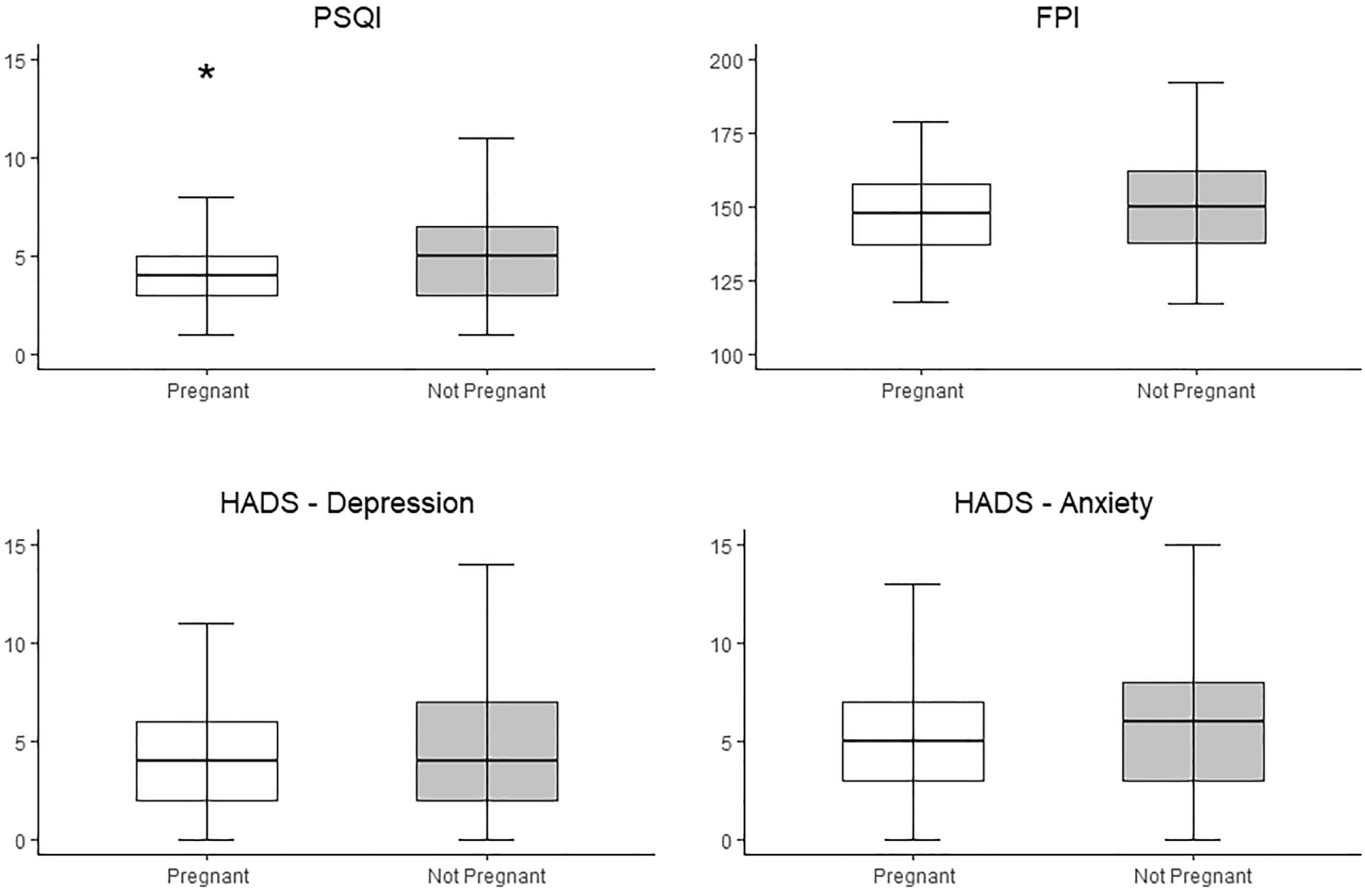
Pittsburgh Sleep Quality Index (PSQI—*upper left panel*), Fertility Problem Inventory (FPI—*upper right panel*), Hospital Depression (*lower left panel*) and Anxiety (*lower right panel*) Scales (HADS) in women who did (white boxes) and did not achieve pregnancy (grey boxes). Data is reported using box plot representation because they were not normally distributed. A statistically significant difference emerged for the PSQI, as highlighted by the asterisk (*p* = 0.004).

PSQI, FPI and HADS scales are presented in Table 3. For PSQI and HADS global scores, data are reported both as median [Interquartile range—IQR] of the total score and as a dichotomous variable grouping women according to a specific validated threshold. This grouping could not be done for FPI because of the unavailability of a validated threshold. Data from continuous analyses on global scores are also illustrated in Fig. 2. A statistically significant difference emerged only for the global score of PSQI, the median [IQR] in pregnant and non-pregnant women being 4^3–5^ and 5^3–7^, respectively (*p* = 0.004). Dichotomous analyses based on the validated threshold confirmed this result, showing that women who did not get pregnant were more likely to have poor sleep quality than those who were successful (Table 3). Among the 80 women with PSQI > 5, only 16 conceived (20%), compared to 65 out of the 183 who had a score ≤ 5 (36%). The OR of pregnancy in women with a PSQI > 5 was 0.46 (95% CI: 0.25–0.86, *p* = 0.02). The OR adjusted for age, ovarian reserve (entered in the model as ln of AFC given the non-normality of the variable), smoking, number of previous IVF cycles and indication to treatment was 0.48 (95% CI: 0.25–0.92, *p* = 0.03). Although statistically significant, the number of oocytes was not included in the model because of the collinearity with ovarian reserve biomarkers.

**Table 3:** Sleep quality, anxiety and depression scores in women who did and did not achieve a pregnancy.

Characteristics	Successfull conception N = 81	Failed conception N = 182	*p*
**Pittsburgh sleep quality index (PSQI)**^**a**^			
Subjective sleep quality	1 [1–1]	1 [1–1]	0.35
Sleep latency	1 [0–1]	1 [0–2]	0.01
Sleep duration	0 [0–1]	1 [0–1]	0.12
Habitual sleep efficiency	0 [0–0]	0 [0–1]	0.17
Sleep disturbances	1 [1–1]	1 [1–1]	0.69
Use of sleep medications	0 [0–0]	0 [0–0]	0.04
Daytime dysfunction	1 [0–1]	1 [0–1]	0.10
Total score	4 [3–5]	5 [3–7]	0.004
Total score > 5	16 (20%)	64 (36%)	0.02
**Fertility problem inventory (FPI)**^**b**^			
Social concerns	30 [29–35]	30 [28–35]	0.51
Relationship concerns	30 [25–34]	30 [25–35]	0.96
Sexual concerns	22 [18–28]	23 [19–27]	0.52
Rejection of childfree lifestyle	28 [23–33]	27 [21–32]	0.10
Need for parenthood	36 [31–43]	39 [34–45]	0.03
Total score	148 [137–158]	150 [138–162]	0.47
**Hospital anxiety and depression scale (HADS)**^**c**^			
HADS—Depression	4 [2–6]	4 [2–7]	0.15
HADS—Depression, score > 7	10 (12%)	34 (19%)	0.20
HADS—Anxiety	5 [3–7]	6 [3–8]	0.22
HADS—Anxiety, score > 7	14 (17%)	53 (29%)	0.05

Data is reported as number (%) or Median [interquartile range], as appropriate.

^a^Data is available for 79 pregnant women and 180 not pregnant women.

^b^Data is available for 79 pregnant women and 176 not pregnant women.

^c^Data is available for 81 pregnant women and 182 not pregnant women.

Finally, correlations between PSQI and the other psychological variables were examined. The Spearman correlation coefficients with FPI, HADS anxiety and HADS depression were 0.19 (*p* = 0.01), 0.33 (*p* < 0.001) and 0.40 (*p* < 0.001), respectively.

## Discussion

The aim of this study was to examine whether sleep quality and psychological health (i.e., infertility related distress and symptoms of anxiety and depression) of women initiating IVF were associated with the chances of success of the treatment. Our findings revealed that only sleep quality but not psychological health was associated with lower chances of pregnancy. However, considering the whole sample, statistically significant correlations were found between sleep quality and psychological wellbeing (either anxiety/depression symptoms or infertility related distress), mirroring the findings of other studies in women with gynecological disorders^27^.

To our knowledge, this is the first study documenting an association between sleep quality and IVF success. To note, the frequency of low sleep quality (PSQI > 5) among women included in our study is substantial, being 30% (80 out of 263 included subjects), a remarkably high rate when compared to local investigations in the general population reporting a 5% prevalence^28^. This high rate of poor sleep quality is in line with previous literature for infertility^20,26^.

The association between sleep quality and unsuccessful IVF may be mediated by impaired psychological health. The observation of a positive correlation with the other scores used (FPI and HADS) tends to support this possibility. To note, as mentioned earlier, the impact of stress and anxiety on IVF outcome is debated in the literature, with five studies suggesting a possible association^9–13^, and other three failing to detect any relation^14–16^. Our present contribution should formally be added to the latter. The discrepancies emerging from the literature are difficult to explain. Sample size, cultural influences and the different instruments used to assess psychological health may play a role.

On the other hand, the identification of a negative association of IVF success with sleep quality but not with psychological well-being might suggest a direct detrimental role of impaired sleep quality, rather than an effect mediated by psychological health. However, a causal relation between sleep quality and the chance of live birth cannot be inferred from our findings because of the observational, cross-sectional (rather than longitudinal) design. In general, disentangling the independent effects of sleep quality and psychological health is challenging because the latter influence the former. One may even claim that sleep quality assessment could be a better indicator of psychological health than other scales specifically dedicated to capture this feature. To note, some previous biological findings suggest a direct and causal link between stress hormones and IVF success. An et al. reported that lower serum cortisol and noradrenaline levels are associated with higher pregnancy rate^29^. Similar results were reported for noradrenaline concentrations in urine at embryo transfer^30^. In addition, an independent group reported that women undergoing IVF with clinically significant affective symptoms had a different pattern of cortisol release compared to controls^31^. Accordingly, variations in plasma cortisol release^32^ as well as the modulation in the concentration of noradrenaline in the synaptic space^33^ account for the propensity to develop anxiety and depression in humans. A second possible link could be indirect, through micronutrients such as serum folate or vitamin D. Deficiency in both nutrients confers vulnerability to affective symptoms^34,35^, and both were reported to predict IVF outcome^36,37^. Finally, one may consider the possible role of an increased inflammation, and in particular the reported higher cervicovaginal levels of cytokines belonging to innate immunity (IL-6 and IL-1β) in women experiencing anxiety and stress with respect to healthy subjects^38^. Of note, impaired regulation of inflammation associated with glucocorticoid receptor resistance was identified as a risk factor of poor pregnancy outcomes such as preterm birth^39^. The interesting aspect is that some of these biomarkers are also involved in the behavioural response to stress and in the onset of depressive and anxiety disorders. However, evidence to draw a link between altered neurohormones, micronutrients and inflammatory molecules and reduced chances of live birth in IVF is lacking. We speculate that this milieu could affect uterine peristalsis, which is a neglected but potentially crucial component in ensuring IVF-mediated pregnancies^40,41^. However, more evidence is needed to verify our hypothesis.

Taken together, our findings highlighted that assessing sleep quality in women undergoing IVF is clinically relevant, due to its association not only with outcome of the procedure, but also with women’s overall psychological health. Physicians may straightforwardly identify women with hampered sleep quality (also considering that it can be easily screened in clinical practice with few questions) and could plan a more in-depth assessment. It may also open the door to interventions that may concomitantly improve psychological well-being and IVF success.

Future studies are warranted to test whether improving sleep quality may be of some benefits for the IVF success. Some previous insights on mental health in general contexts offered promising findings. Given the need of supporting mental health of women who intend to undergo IVF, different types of strategies were tested. The use of Selective Serotonin Reuptake Inhibitors (SSRIs) is advisable in women scheduled for IVF and suffering from clinically significant anxiety and depression to increase the probability of achieving pregnancy^42^. Short-term high dose (3 mg/day) melatonin failed to ameliorate sleep quality in women beginning IVF, but it contributed to improve the oocyte and embryo quality^43^. A RCT on the use of melatonin in IVF women with sleep impairment conversely provided disappointing results^44^. Regarding non-pharmacological treatment, mindfulness-based group counselling^45^ and acupuncture^46^ showed to be beneficial in improving stress, depression, and anxiety in women under IVF treatment. Their effect on the chances of pregnancy remains controversial. Studies specifically focussing on improving sleep disturbances are lacking.

Future research should also clarify whether the greater prevalence of bad sleep quality in women with infertility may be also related to unhealthy lifestyles (e.g., excessive amount of work and consequent stress, use of mobile phones during bedtime, lack of physical exercise, unhealthy diet) that are common in contemporary society, as also suggested by Wang et al*.*^47^.

Some limitations of our study should be recognized. The data were collected during treatment, which did not allow to properly investigate the association between sleep quality, infertility, and IVF itself. A control group of women planning or trying to become pregnant could have been of interest. It should also be considered that sleep quality was assessed using a self-reported sleep measure (although validated and extensively used by clinicians and researchers), which does not ensure the objectivity of the observations^27^.

In conclusion, the results of the present research indicate that only altered sleep quality is common in women scheduled for IVF and could influence the success of the procedure. Although more research is needed to disentangle whether a causal relation does exist and what pathogenetic mechanisms are involved, our findings indicate that the assessment of sleep quality throughout the treatment period should be part of the multidisciplinary clinical management of patients undergoing IVF.

## Methods

Women undergoing IVF treatment at Infertility Unit, Fondazione IRCCS Ca’ Granda Ospedale Maggiore Policlinico, Milan (Italy) between September to December 2019, were prospectively and consecutively enrolled for the purpose of the study. The protocol of this study was approved by the local Ethical Committee (Comitato Etico Milano Area 2, N 494_2019). Patients provided a written informed consent to participate, and all research was performed in accordance with the Declaration of Helsinki.

Inclusion criteria were admission for oocyte retrieval. Exclusion criteria were neurologic or severe psychiatric disorder (diagnosis of Major Depressive Disorder, Bipolar Disorder or Schizophrenia Spectrum Disorders) and obstructive sleep apnea syndrome, as well as other anatomical factors that can reduce embryo implantation (hydrosalpinx, uterine malformations, endometrial polyps, and submucosal fibroids). We also excluded women who did not speak fluent Italian, as the questionnaire used is validated for Italian speaking individuals.

The Information about the following baseline variables was collected: age, education, job status, smoking status, Body Mass Index (BMI), duration of infertility (months), previous deliveries, serum FSH (IU/mL), serum Anti-Müllerian Hormone (AMH) (ng/mL), antral follicle count (AFC), indication for IVF. In addition, we collected data on pregnancy outcome and on cumulative live births. This information was obtained through an active and systematic follow-up, as described elsewhere^48^.

The following rating scales were administered to assess women’s mental health: the Pittsburgh Sleep Quality Index (PSQI), the Fertility Problem Inventory (FPI), and the Hospital Anxiety and Depression Scale (HADS).

PSQI assesses the sleep quality in the last month. Nineteen individual self-rated items make up seven “component” scores: subjective sleep quality, sleep latency, sleep duration, habitual sleep efficiency, sleep disturbances, use of sleeping medication, and daytime dysfunction. The sum of scores for these seven components yields one global score ranging from 0 (optimal sleep quality) to 21 (the poorest sleep quality)^49^. A score > 5 represents the cut off for bad sleepers^50^. The survey was validated in Italian^28^. Evaluation of sleep quality was exclusively done with the PSQI questionnaire; no attempt was made to actively investigate particular sleep disturbances.

FPI assesses infertility-related distress validated in Italian^51,52^. This tool consists of 46 items grouped in five different domains: (1) social, (2) relationship and (3) sexual concerns, and (4) rejection of child-free lifestyle and (5) need for parenthood. Participants are asked to rate how much they agree or disagree with fertility-related concerns or beliefs and score ranges from 0 (strongly disagree) to 6 (strongly agree). The overall score ranges from 46 to 276, and a higher score indicates higher perceived fertility-related stress. A validated threshold to distinguish women experiencing an excessive distress from those who do not is not reported in the literature and was thus not used in this study.

HADS measures the severity of anxiety and depression. The HADS, largely administered to assess affective symptoms in patients with medical comorbidities or in healthy subjects referring to hospitals, consists of two subscales (one for depression and the other for anxiety), each one consisting of seven items. Scoring from each item ranges from 0 to 3, thus the total score for each subscale can range from 0 to 21. A score > 7 in each subscale denotes the presence of clinically significant anxiety and depression^53^. The questionnaire was validated in Italian^54^.

Data on clinical variables were collected by charts, while the rating scales were administered on the day of oocyte retrieval since on that day patients spent many hours at the center and had sufficient time to give all the needed information. Pregnancy assessment was performed testing serum human Chorionic Gonadotropin (hCG) at + 14 days after oocytes retrieval (adapted for frozen cycle). Women found to be pregnant were scheduled a second serum assessment 48–72 h later and, if appropriate, they underwent transvaginal sonography two weeks later (thus 4 weeks after ET). Clinical pregnancy was defined as the observation of an intrauterine gestational sac containing a viable fetus at 6+ weeks’ gestation.

Statistical analyses were performed through The Statistical Package for Social Sciences (SPSS) for Windows (version 26.0). Data were presented as number (%), mean ± Standard Deviation (SD) or median [Interquartile Range—IQR], as appropriate. Women who did and did not achieve clinical pregnancy were compared for the collected quantitative and qualitative variables using Fisher’s exact tests, χ2 tests, unpaired Wilcoxon tests, or unpaired Student’s t tests, as appropriate. Variables found to be non-normal at Kolmogorov test were reported as median [IQR] and compared using nonparametric statistics. Women who did not fully complete one of the questionnaires were excluded in the analysis of the specific questionnaire. Multivariate logistic analyses were performed to capture the independent role of the psychological variables found to significantly differ at univariate analyses. In this model, we included age, antral follicle count (AFC), number of previous IVF cycles, indication to treatment and other baseline variables found to differ between women who did and did not achieve pregnancy. Continuous variables not normally distributed were ln transformed before being entered in the multivariate model. Spearman correlation index was used to assess possible correlations between the scores.

The sample size was estimated focusing on sleep quality (PSQI > 5). Previous local assessments showed that this rate is about 5% in the general population^28^. Given the emotional burden of IVF, we hypothesized that it could be more than 10% in our population and deemed of clinical relevance demonstrating a 2.5 folds increase in women who failed to become pregnant (25% versus 10% in pregnant women). Considering an expected live birth rate based on local data of about 30% and setting type I and II errors at 0.05 and 0.20, the total number of women to be included was about 250 (Fleiss method with continuity correction).

## Data Availability

The datasets generated and analysed during the current study are not publicly available due to reasons of sensitivity (human data) but are available from the corresponding author on reasonable request.
